# Comparison of the Efficacy of Two Novel Antitubercular Agents in Free and Liposome-Encapsulated Formulations

**DOI:** 10.3390/ijms22052457

**Published:** 2021-02-28

**Authors:** Nikoletta Kósa, Ádám Zolcsák, István Voszka, Gabriella Csík, Kata Horváti, Lilla Horváth, Szilvia Bősze, Levente Herenyi

**Affiliations:** 1 Department of Biophysics and Radiation Biology, Semmelweis University, 1094 Budapest, Hungary; nikole.kosa@gmail.com (N.K.); zolcsak.adam@med.semmelweis-univ.hu (Á.Z.); voszka.istvan@med.semmelweis-univ.hu (I.V.); csik.gabriella@med.semmelweis-univ.hu (G.C.); 2 MTA-ELTE Research Group of Peptide Chemistry, Eötvös Loránd University, Hungarian Academy of Sciences, 1518 Budapest, Hungary; khorvati@gmail.com (K.H.); lilla.horvath@yahoo.com (L.H.); 3 Institute of Chemistry, Eötvös Loránd University, 1518 Budapest, Hungary

**Keywords:** coumaran, 2,3-dihydrobenzofuran, pH-sensitive stealth liposome, monocomponent liposome, dynamic light scattering, absorption spectrometry, flow cytometry, colony-forming units

## Abstract

Tuberculosis is one of the top ten causes of death worldwide, and due to the appearance of drug-resistant strains, the development of new antituberculotic agents is a pressing challenge. Employing an in silico docking method, two coumaran (2,3-dihydrobenzofuran) derivatives—TB501 and TB515—were determined, with promising in vitro antimycobacterial activity. To enhance their effectiveness and reduce their cytotoxicity, we used liposomal drug carrier systems. Two types of small unilamellar vesicles (SUV) were prepared: multicomponent pH-sensitive stealth liposome (SUV_mixed_) and monocomponent conventional liposome. The long-term stability of our vesicles was obtained by the examination of particle size distribution with dynamic light scattering. Encapsulation efficiency (EE) of the two drugs was determined from absorption spectra before and after size exclusion chromatography. Cellular uptake and cytotoxicity were determined on human MonoMac-6 cells by flow cytometry. The antitubercular effect was characterized by the enumeration of colony-forming units on *Mycobacterium tuberculosis* H_37_Rv infected MonoMac-6 cultures. We found that SUV_mixed_ + TB515 has the best long-term stability. TB515 has much higher EE in both types of SUVs. Cellular uptake for native TB501 is extremely low, but if it is encapsulated in SUV_mixed_ it appreciably increases; in the case of TB515, quasi total uptake is accessible. It is concluded that SUV_mixed_ + TB501 seems to be the most efficacious antitubercular formulation given the presented experiments; to find the most promising antituberculotic formulation for therapy further in vivo investigations are needed.

## 1. Introduction

**Figure 1:**
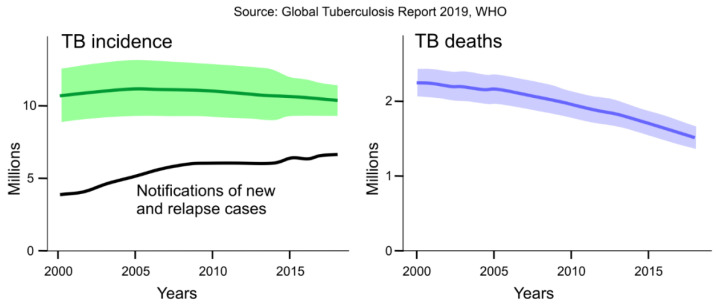
Global trends in the estimated number of incident tuberculosis (TB) cases with the new and relapse occurrences and the estimated number of deaths caused by TB, 2000–2018. (Shaded areas represent uncertainty intervals.)

Tuberculosis (TB), an ancient infectious disease with worldwide occurrence, is caused by the intracellular pathogen *Mycobacterium tuberculosis* (*Mtb*). According to the newest World Health Organization (WHO) report, while the TB mortality rate is falling at about 3% per year, in 2018 more than 1.5 million deaths were attributed to the disease [1]. Figure 1 shows this decrease in the estimated number of deaths caused by TB in the 2000–2018 interval but also indicates that the estimated number of incident TB was practically unchanged in the same interval. In 2018 still, about 10 million people globally were infected with TB; moreover, the notifications of new and relapse cases persistently increased. Treatment of TB is a combination of four first-line drugs [2,3,4] and takes at least six months—much longer compared with other bacterial infections because *Mtb* bacteria are capable of surviving and growing inside the macrophages [5]. Patient adherence to therapy is far from optimal due to the lengthy treatment and unwanted side effects of medicines [3,6]. Insufficient patient adherence is the main reason behind the high number of appearing resistant TB strains. The WHO reported about half a million rifampicin and multidrug-resistant TB cases in 2018; from these cases more than 200,000 people died [1]. Multidrug-resistant TB (MDR-TB) means that strains are resistant not only to rifampicin (RIF) but also to isoniazid (INH). The other class of TB resistance is the extensively drug-resistant (XDR-TB) [4]. In this instance, strains are resistant to both RIF and INH as well as one of the fluoroquinolones and at least one of the three injectable second-line antituberculosis drugs (amikacin, capreomycin, and kanamycin) [7]. The highest occurrence of MDR-TB or XDR-TB—more than 25%—was reported in India [1]. Therefore, based on these facts, the development of new potent drugs against *Mtb* is of utmost importance.

The identification of potential protein targets on *Mtb* that are different from host cell proteins may provide opportunities for the development of *Mtb* selective inhibitors and ultimately effective new drugs. Therefore, biochemical mechanisms of *Mtb* were analyzed to find targets unique and essential to that microorganism, and one possible target was identified. The *Mtb* dUTPase enzyme, which is required for the growth of the bacteria, is a promising target enzyme [8,9,10,11]. The dUTPase plays an important role in the preventive DNA repair mechanism, and its X-ray crystal structure and catalytic mechanism have been published [12,13]. It has been proposed that a drug binding to the species-specific loop of dUTPase might be an effective tool to inhibit the growth of *Mtb* [11,14].

In silico docking is one of the most effective approaches for identifying drug-like ligands/novel small molecule inhibitors to protein targets of known 3D structure. The new FRIGATE docking software of Uratim Ltd. [15,16] applied a novel hybrid approach as described by Horváti [17] and Scheich [16] and their coworkers earlier. Libraries of commercially available molecules (ZINC database) [18,19] were docked to the target dUTPase enzyme (2PY4) [11] using the FRIGATE docking method.

The best hits were filtered according to Lipinski’s rules [20], which evaluate drug-likeness. 

More than fifty percent of the tested molecules showed relevant Minimum Inhibitory Concentration (MIC) (lower than 100 μg/mL). After considering all the points of view, two coumaran (2,3-dihydrobenzofuran) derivatives, both members of the TB5 family, were selected in the recent past for further research: TB501, and TB515 (see Figure 5 and Table 7) [17,21,22,23]. 

The intracellular concentration of orally administered antitubercular drugs—crucial for successful therapy—is unsatisfactory in most cases. Increasing dosage can cause patient non-compliance because of the emerging side effects. Biodistribution of antituberculous drugs like RIF and INH can be enhanced by nanocarrier systems (liposomes, niosomes, solid lipid nanoparticles, micelles) which are widely reported in the literature as a possibility for enhancing TB therapy, carrying several advantages like biodegradability, non-toxicity and reduction of side effects [24,25]. Lung specificity and intracellular delivery of nanocarriers can be reached by well-selected components and surface modification, like conjugation of peptides, antibodies, transferrin, and lectins. The most commonly applied components are phosphatidylcholine, cholesterol, distearoylphosphatidylethanolamine-polyethylene glycol, and dicetyl-phosphate to lung targeting [5,24,25,26,27].

Liposomes are excellent nanocarrier systems. The application of this unique formulation method has several enticing advantages, such as enhancing the biodegradability of the encapsulated molecules. Liposomal drug formulation makes it achievable to incorporate substances with vastly different physicochemical properties, most importantly with vastly different water solubility. Several liposomal formulations are on the market and are continuously being used by clinicians, for example Doxil^®^, Vysudine^®^, DepoCyte^®^ and Amphotec^®^ [28,29]. The development of novel targeting schemes to improve the therapeutic index of drugs encapsulated within liposomes is widely reported in the literature. According to Paliwal and coworkers [30], among the most frequently used nanocarriers are pH-sensitive liposomes. Such liposomes have been used as an alternative to conventional liposomes. They are explicitly designed to release their contents in response to the acidic pH of the endosomal system. pH-sensitive liposomes are generally composed of dioleoylphosphatidyl-ethanolamine (DOPE) and cholesteryl hemisuccinate (CHEMS). A major limiting factor for the broad application of pH-sensitive liposomes is their recognition by the phagocytes of the reticuloendothelial system [30]. The surface-modified targeted liposomes are capable of bypassing barriers imposed by the biological environment, even up to cellular and sub-cellular levels. A significant step in the development of these liposomes came with the inclusion of the synthetic polymer polyethylene glycol (PEG) in liposome composition. Due to the presence of PEG on the surface of the liposomes, the uptake of the PEGylated liposomes is strongly diminished by the mononuclear phagocyte system. It is for this reduced immunogenicity that the PEGylated liposomes are called stealth liposomes [31,32].

Accordingly, for the encapsulation of antitubercular agents, we applied two liposomal formulations. One of them is a complex vesicular system, which consists of DOPE, CHEMS, and pegylated distearoylphosphatidylethanolamine (DSPE-PEG) as a multicomponent pH-sensitive stealth liposome, referred to as small unilamellar vesicles (SUV_mixed_), and the other one is a monocomponent conventional liposome from dipalmitoylphosphatidylcholine (DPPC) lipid (called as SUV_DPPC_) as a reference. 

The main goal of the present study incorporating the compounds into liposomal nanocarrier systems was to enhance cellular uptake and to improve the therapeutic efficacy of the intracellular population of the bacteria. Since *Mtb* is an intracellular pathogen, proposedly a liposomal formulation of the drug candidates can achieve enhanced cellular uptake and grant molecular protection to the active agents by encapsulating them inside a drug delivery system, protecting them from unwanted harm before reaching the site of action [5].

This work focuses on the physicochemical characterization of liposomal formulations. Based on the information provided by the measurements, one can determine which formulation may serve as the most promising antituberculous treatment modality from our drug candidates.

## 2. Results

### 2.1. Physicochemical Characterization of Liposome Samples

#### 2.1.1. Homogeneity and Long-Term Stability

**Figure 2:**
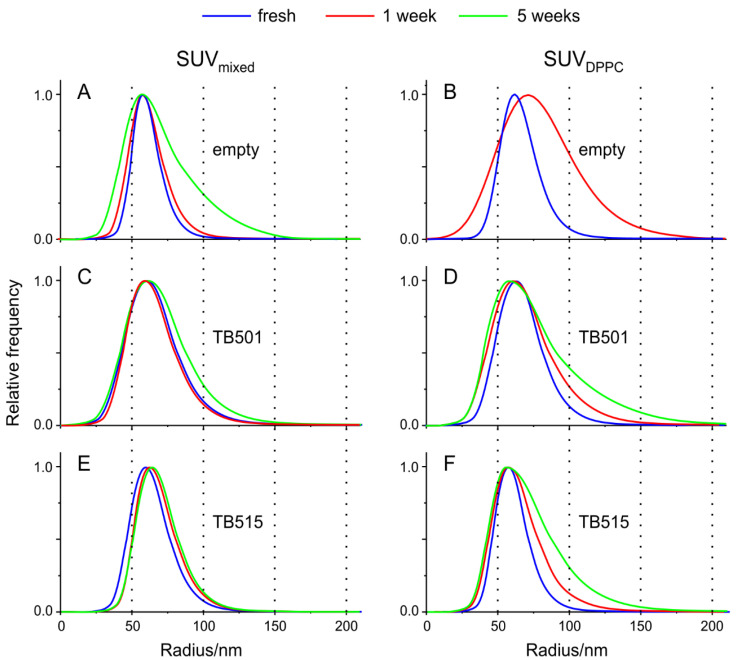
(**A**–**F**) Homogeneity and long-term stability of liposomes are measured by dynamic light scattering (DLS). The size distribution of the two types of small unilamellar vesicles (SUVs) (SUV_mixed_ and SUV_DPPC_) without and with the two antitubercular agent candidates separately (TB501 and TB515): blue curve freshly prepared; red after one week; green after five weeks (in the case of empty SUV_DPPC_ after five weeks, the DLS autocorrelation function could not be evaluated).

We prepared altogether six different types of liposome samples from the two types of SUVs (SUV_mixed_ and SUV_DPPC_) without and with the two antitubercular agent candidates separately (TB501 and TB515). Figure 2 shows the size distribution of freshly prepared SUVs and the same samples after one and five weeks. The estimated mode (most probable value) of the radius of the freshly prepared vesicles is about 60 nm with a small deviation for all the samples. This result is in accordance with the anticipated size of the vesicles because the pore size of the polycarbonate filter of the extruder was 100 nm. This parameter increases about 15% after one week in the case of empty SUV_DPPC_, but the other formulations remain practically unchanged. This tendency continued, and after five weeks the measured data could not be evaluated.

The other most crucial parameter of the curves is the full width at half-height (FWHH), which is—as a function of elapsed time—also characteristic of the stability of liposomes. While in the beginning, the empty SUV_mixed_ had the shortest FWHH parameter (22 nm) (meaning it was the most homogeneous sample), this doubled after five weeks. Based on these data, we have three critical observations: (i) Antitubercular agents generally have a stabilization effect for all the liposomal systems; (ii) SUV_mixed_ is more stable than SUV_DPPC_; and (iii) TB515 causes the narrower FWHH parameter (25–30 nm versus 35–40 nm). This indicates a more homogeneous distribution.

#### 2.1.2. Encapsulation Efficiency

**Figure 3:**
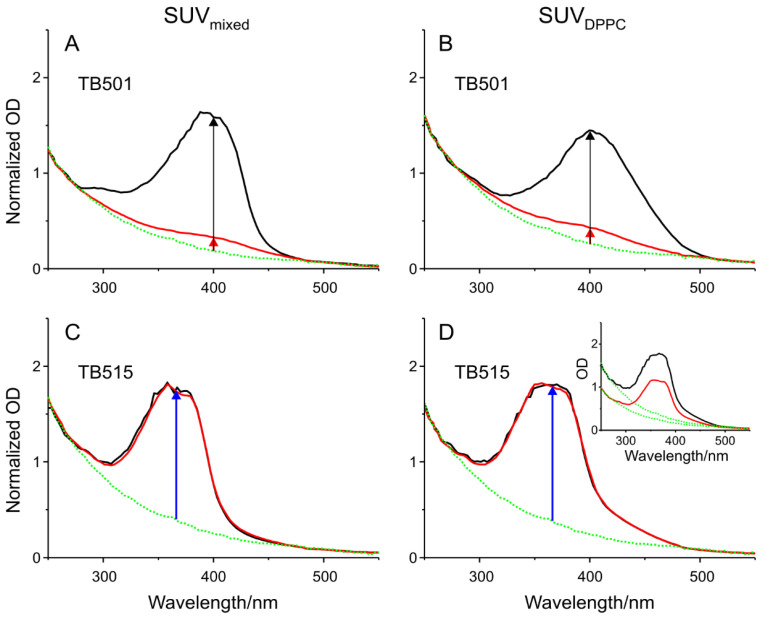
(**A**–**D**) Determination of encapsulation efficiency (EE). Absorption spectra of the encapsulated antitubercular agent candidates (TB501 and TB515) in the two different SUV formulation (SUV_mixed_ and SUV_DPPC_), represented with normalized (optical density (OD) before (black curves) and after (red curves) size exclusion chromatography (SEC). Green curves show the signal of the scattered light of liposomes themselves. These curves were used to calibrate the normalization of OD. The inset in part D shows the original spectra without normalization.

Besides long-term stability, another important feature of drug carriers is their EE. As it is written in the Materials and methods section, SEC was used to separate the encapsulated drug from free molecules. The measured absorption spectra can be seen in Figure 3, but the inset in part D shows an original spectrum before and after the SEC without normalization.

We repeated all the measurements and obtained the real absorbances around 400 nm in the case of TB501 and around 370 nm in the case of TB515 without the scattered light signal according to the arrows in Figure 3. From these data based on the proportionality between the absorbance and concentration, EE was calculated in all four cases and summarized in Table 1.

It is conspicuous that while in the case of TB515 the EE is almost 100%, but in the case of TB501, it reaches a value just above 10%. This effect can probably be explained primarily by the very different sizes and side chains of the two molecules. The deviations of EE between the two different SUVs are practically negligible, but SUV_DPPC_ has slightly higher values.

### 2.2. In Vitro Cellular Uptake and Cell Viability 

Determination of cell viability and internalization profile is essential in evaluating the in vitro response for different compounds and delivery constructs. Flow cytometry and fluorescent microscopy are reliable methods for studying these features. 

Table 2 shows the ratio of living cells (%) for native agents and liposomal formulations.

It is observable that the untreated cells (control) show practically the same results (89–95% of the cells remained living). It is also clearly observable that the encapsulation unambiguously decreases the cytotoxicity (increases the ratio of living cells), and that in this aspect the SUV_DPPC_ is a more appealing choice.

**Figure 4:**
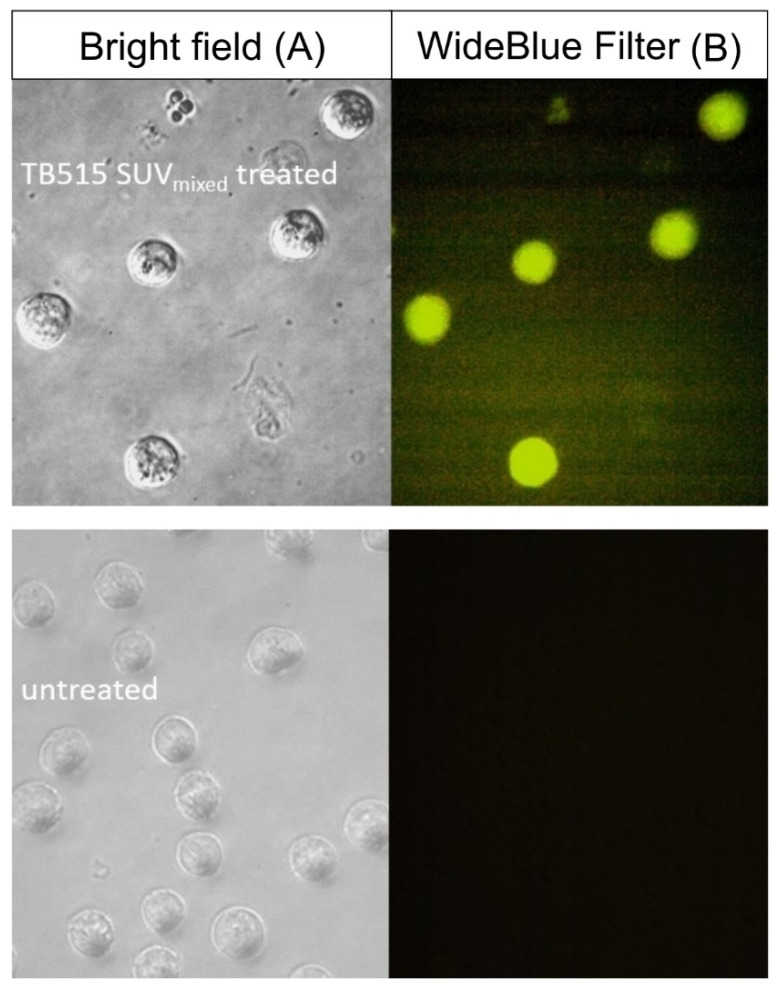
Internalization of TB515 antitubercular compound encapsulated in SUV_mixed_ by MonoMac-6 human monocytic cells captured by fluorescence microscopy. Column (**A**) displays bright-field images of untreated control cells and treated cells (c = 25 μM; 3 h) Column (**B**) displays untreated and treated cells (c = 25 μM; 3 h) imaged with WideBlue Filter.

In the case of TB515 encapsulated in SUV_mixed_ a contrasted and detailed fluorescent microscopy image could be produced, which indicates a highly efficient cellular uptake by MonoMac-6 cells (see Figure 4). 

Similar results are presented by flow cytometry in quantitative form using the fluorescence signal. Table 3 shows the cellular uptake rate of the two drug molecules in native and in encapsulated form. Due to the enormous difference in uptake efficiency, we could not characterize it in the same way. While the 100% uptake rate was reachable for TB515 with native and both encapsulated agents below 160 μM concentration, in the case of TB501 at the same agent concentration the maximal uptake rate was less than 2%. Nevertheless, an unambiguous increase in cellular uptake rate is observable due to encapsulation, especially in the case of SUV_mixed_.

### 2.3. Intracellular Efficacy on Infected Host Cells

The in vitro antituberculous effect of drug candidates was studied by counting the CFU on *Mtb* H_37_Rv infected MonoMac-6 cell cultures, which were treated by native or encapsulated agents and incubated for four weeks. The acquired data are presented in Table 4 at different concentrations for both agents.

It is clearly observable that the native TB515 is ineffective at this concentration range, and that the TB501 encapsulated in SUV_mixed_ seems to be the most effective antitubercular agent candidate.

TB515 displayed lower MIC values for both extracellular *Mtb* H_37_Rv and MDR A8 cultures. Both TB515 and TB501 have displayed negligible cytotoxic activity on MonoMac-6 cells. They exhibited favorable selectivity towards bacteria. Their selectivity indexes are 4.0 and 4.7 (see Table 5).

## 3. Discussion

In evaluating the results, we tried to find the optimal, most promising antitubercular agent candidate for therapy.

The ultimate target of an antitubercular drug candidate is mainly intracellular (inside certain host cells), and the interaction of the compounds with the cellular/bacterial membrane is expected to affect the compound’s bioavailability and efficacy [5]. The chemical nature of the antituberculous compound determines the level of entrance into host cells, and even small differences in their chemical structure can have a remarkable influence on their lipophilicity, their capacity to cross cell membranes, to accumulate intracellularly, and the eventual antitubercular effect.

Free compounds (TB501 and TB515) have activity on extracellular bacteria, but limited efficacy on the intracellular population due to their poor cellular uptake rate. The main goal was to enhance cellular uptake and intracellular activity to inhibit bacteria in the host cell environment. To achieve increased compound transport across the plasma membrane and to improve the permeability of the antitubercular agent (free compounds), we constructed liposomal nanocarrier systems. The cellular uptake of the drug candidates was monitored in vitro with fluorescent microscopy. We followed the physicochemical changes which resulted from the incorporation of our drug candidates in our liposomal systems.

Considering the long-term stability, according to the relative broadening (%) of size distribution after the 5th week we determined the long-term stability order as: SUV_mixed_+TB515 (≈5%) < SUV_mixed_ + TB501 (≈20%) < SUV_DPPC_ + TB501 (≈50%) < SUV_DPPC_ + TB515 (≈80%). As it is visible in Figure 2, the broadening is unidirectional. The mode of the particle sizes increased in all the liposomes, thus we can assume that the origin of this change is the resulting vesicle fusion.

The total effective drug content of a liposomal formulation depends on the encapsulation efficiency as well as its cellular uptake. In the case of TB515, both characteristic parameters are quasi identical for the two types of liposomes. In the case of TB501, taking into consideration both parameters, the total effective drug content for SUV_DPPC_ is about four times smaller than for SUV_mixed_, but it is at least 70 times less than what it would be with TB515 content.

Based on the flow cytometry measurements, we may conclude that the encapsulation significantly decreases the cytotoxicity for both agents in either SUV environment. From this aspect, SUV_DPPC_ is more effective than SUV_mixed_. It must be mentioned that the decrease in cell viability in the case of native agents is not more than 25%.

Comparing the in vitro intracellular antitubercular efficacy, we must take into consideration the total effective drug content of the liposomal formulations. As a first step, we may compare the native agents. TB501 starts to show some decrease of CFU at 25 μM concentration, whereas TB515 remains ineffective up to 100 μM even though the cellular uptake can reach hundreds of times higher. Among the liposomal formulations, SUV_mixed_ + TB501 is the most effective. It can cause the same decrease of CFU with an agent concentration of four times less, besides having about 70 times lower cellular uptake compared to SUV_mixed_ + TB515. SUV_DPPC_ + TB501 and SUV_DPPC_ + TB515 proved to be somewhat less effective compared to SUV_mixed_ formulations.

To place this in context with our results, the ability to inhibit intracellular bacteria and the selectivity towards the host MonoMac-6 cells, the efficiency of the studied agents against intracellular bacteria can not be attributed to their cytotoxicity towards the host cells. Free and encapsulated TB501 molecule shows better effectiveness on infected MonoMac-6 cell cultures despite their higher MIC values compared to the TB515 drug candidate. That could be explained by the vastly different intracellular metabolic pathways of TB501 and TB515 molecules.

In Table 6, we summarized all the previous results according to a qualitative scale. Based on this, we evaluate the most promising antitubercular agent formulations.

It is clearly seen from the last columns for each candidate in this table that the liposomal formulation can provide significant improvements. Another important observation is that even though the total effective drug content of both SUV + TB501 liposomal formulations is extremely low compared to both SUV + TB515 formulations, their antitubercular effect is more pronounced.

If we compare the beneficial features of the two different SUVs, we may highlight the advantages that SUV_mixed_ is a pH-sensitive and stealth liposome. In our SUV_mixed_ system, the molar ratio of PEG is about 2.5%. Based on scientific literature the shielding effect of PEG can be increased by increasing its load. The maximum shielding effect can be reached at about 10% PEG content, when PEG forms a dense, brush-like coating [31]. On the other hand, many studies have reported that unexpected immune responses have occurred against PEG-conjugated nanocarriers [33]. Additionally, the measured intracellular inhibition is higher in the case of encapsulated TB501 even at extremely low drug concentrations.

## 4. Materials and Methods 

### 4.1. Chemicals

**Figure 5:**
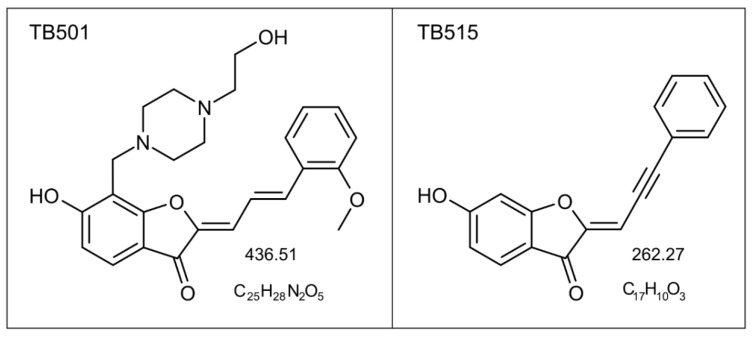
Chemical structure of the two in silico identified small molecular weight trisubstituted coumaran (2,3- dihydrobenzofuran) antitubercular agent candidates: TB501 and TB515.

**TB501**: 6-hydroxy-7-{[4-(2-hydroxyethyl)piperazin-1-yl]methyl}-2-[(2E)-3-(2-methoxyphenyl)prop-2-en-1-ylidene]-2,3-dihydro-1-benzofuran-3-one; and **TB515**: (2E)-6-hydroxy-2-(3-phenylprop-2-yn-1-ylidene)-2,3-dihydro-1-benzofuran-3-one compounds, which are derivatives of in silico-identified potential antibacterial agents [17,21,34] were purchased from SONEAS Research Ltd. (formerly Ubichem Research Ltd.) Budapest, Hungary. The chemical structure and chemical features of TB501 and TB515 molecules can be seen in Figure 5 and Table 7.

1,2-dipalmitoyl-sn-glycero-3-phosphocholine (16:0) (DPPC), 1,2-dioleoyl-sn-glycero-3-phosphoethanolamine (18:1) (DOPE) and 1,2-distearoyl-sn-glycero-3-phosphoethanolamine-N-[methoxy(polyethylene glycol)-2000] (ammonium salt) (DSPE-PEG) phospholipids were obtained from Avanti Polar Lipids Inc. (Alabaster, Alabama, USA). Other chemicals such as sodium chloride (NaCl), sodium hydrogen phosphate (Na_2_HPO_4_), chloroform (CHCl_3_), dimethyl sulfoxide (DMSO), cholesteryl hemisuccinate (CHEMS), a cholesterol derivate were acquired from Sigma-Aldrich (Budapest, Hungary). Antibacterial agents were dissolved in DMSO or directly added to lipids at the beginning of SUV preparation. All materials used for in vitro experiments were suitable for cell culture (bioreagent grade and tested), namely: RPMI-1640, fetal bovine serum (FBS), glutamine (Lonza, Basel, Switzerland), trypsin, and gentamicin (Sigma-Aldrich, Budapest, Hungary). The HPMI (HEPES buffered RPMI) buffer was prepared and sterile-filtered in our laboratory using components obtained from Sigma-Aldrich: analytical grade (NaCl, KCl, MgCl_2_, CaCl_2_, NaHCO_3_, Na_2_HPO_4_), 4-(2-hydroxyethyl)piperazine-1-ethanesulfonic acid (HEPES), and glucose (cell culture grade) [35].

### 4.2. Preparation of Liposomes

Two types of SUVs were formulated by the thin film hydration method [36]. The multicomponent pH-sensitive stealth liposome was made from a mixture of DOPE, CHEMS, and DSPE-PEG at a 5:4:1 mass ratio (SUV_mixed_). The ratio of components written in the literature by Simões and coworkers [37] was slightly modified. The monocomponent reference liposomes were made from DPPC (SUV_DPPC_).

First, a thin lipid layer was produced on the wall of a glass vial—solid lipids (and drugs) were dissolved in chloroform (10 mg with 10:1 lipids-drugs molar ratio in 100 µL) and then dried with a gentle stream of nitrogen gas to remove the organic solvent. Afterward, it was kept in a desiccator at least overnight. 

The lipid film was hydrated with 1 mL phosphate-buffered saline (PBS) (10 mM phosphate, 137 mM NaCl, pH 7.4) at 55 °C. In order to sterilize the buffer Millipak membrane filter with 0.22 µm was used. The formulation of the SUVs was achieved by the extrusion technique. We used a thermostated (55 °C) extruder (Avanti Polar Lipids, Inc., Alabaster, AL, USA, Mini-Extruder). The lipid suspensions were pressed 41 times through a polycarbonate filter (Whatman) of 100 nm pore size. 

For the determination of size distribution by dynamic light scattering (DLS), a volume of 40 µL of liposome sample was diluted with 200 µL of PBS buffer.

For absorption measurements, we used the samples without dilution to achieve a better signal-to-noise ratio.

### 4.3. Dynamic Light Scattering 

We used the same dynamic light scattering (DLS) equipment, which was uniquely constructed and described in our earlier publication [38]. Briefly, the light of a diode-pumped solid-state (DPSS) laser light source (Melles Griot 58-BLS-301, 457 nm, 150 mW) scattered by the samples was detected at 90° by a Hamamatsu light detector (H7155 PMT module). The autocorrelation functions were analyzed by the maximum entropy method (MEM) [39]. Finally, we used the weighting factor of *r*^−2^ (*r* is the radius), and the relative frequency distribution of the hydrodynamic radius was determined.

### 4.4. Size Exclusion Chromatography and Absorption Spectrometry

Size exclusion chromatography (SEC) was used to separate the encapsulated drug from free molecules [40]. A column with 5.8 cm^3^ volume and 0.7 cm diameter was filled with Sepharose 4B gel. Before pouring it into the column, the gel was swollen in PBS buffer overnight. The gel was saturated with an empty liposome sample to avoid retention [33,41]. After loading the sample into the column, we collected 0.5 mL fractions.

We measured the absorption spectra of different fractions with a NanoDrop 1000 spectrophotometer (Thermo Fisher Scientific, Wilmington, Delaware). The applied sample volume was 2 µL. Absorbance was recorded before and after SEC as a function of wavelength in the 250–550 nm range. We determined the encapsulation efficiency (EE) based on the absorption spectra measured before the SEC procedure, and later on some initial fractions. We also measured the signal of the scattered light of empty liposomes themselves (without antitubercular agents) in the same wavelength range and it was fitted by a power function with an exponent close to −4. We applied a normalization procedure based on the scattered light signal of SUVs. The normalization factor was determined by seeking the smallest mean square displacement of the two curves. Thus considering Beer’s law, virtually the total liposome concentrations of the samples became equivalent. EE was evaluated from these measured data.

### 4.5. Assessment of In Vitro Cellular Uptake and Cytotoxicity Using Flow Cytometry

Due to their intrinsic fluorescent property, TB501 and TB515 compounds can be used conveniently for measurements by flow cytometry and imaging by fluorescent microscopy [17,23]. Thus in vitro cellular uptake and cytotoxicity of liposomal compounds and non-encapsulated drugs were determined by flow cytometry on MonoMac-6 human monocytic cell line (DSMZ no.: ACC 124, Deutsche Sammlung von Mikroorganismen and Zellkulturen GmbH, Braunschweig, Germany) [42], similarly as described earlier [16]. Human MonoMac-6 cells were chosen as an in vitro model for investigating uptake and cytotoxicity. This culture represents monocytic cells with a closely related pattern of surface, phenotypic, functional features, and adhesion properties of mature monocytes and macrophages to the main host cells for intracellular pathogen *Mtb*. Briefly, MonoMac-6 cells were maintained as an adherent culture in RPMI-1640 media supplemented with 10% heat-inactivated fetal calf serum (FCS) L-glutamine (2 mM) and gentamicin (35 μM) at 37 °C in a humidified atmosphere containing 5% CO_2_. Cells were harvested in the logarithmic phase of growth and plated on a 24-well tissue culture plate (10^5^ cells/1 mL medium/well) 24 h prior to the experiment. Compounds were dissolved in serum-free (SFM) RPMI-1640 medium, and serial dilutions were prepared. The highest concentration of the compounds on the cells was 320 μM. That was the highest concentration of TB515 molecule that was encapsulated by liposomes. Cells were incubated with compounds for three hours (37 °C, 5% CO_2_ atmosphere). After washing twice with RPMI medium, supernatants were removed and 100 μL 0.25% trypsin was added to the cells. Two minutes of incubation with trypsin was followed by the addition of 0.8 mL 10% FCS/HPMI (HPMI: 100 mM NaCl, 5.4 mM KCl, 0.4 mM MgCl_2_, 0.04 mM CaCl_2_, 10 mM HEPES, 20 mM glucose, 24 mM NaHCO_3_ and 5 mM Na_2_HPO_4_ at pH = 7.4 [35]. Then cells were washed and re-suspended in 0.25 mL HPMI.

Cellular uptake and cell viability were determined by using a BD LSR II flow cytometer (BD Biosciences, San Jose, CA, USA) with a 488 nm (Coherent Sapphire, 22 mW) laser for measuring the intrafluorescence signal (channel FITC LP505; emission at λ = 505 nm; LP 505, BP 530/30, excitation λ = 488 nm) and the forward and side scattered (FSC, SSC) light intensity. Data were analyzed with FACSDiva 5.0 software. All measurements were performed in duplicate, and the mean fluorescent intensity and percentage of FITC positive (compound containing) cells were presented. To assess relative viability, the percentage of live cells was compared to untreated control cells. For comparison, we used the samples with 160 μM compound concentration. Parallel with flow cytometry measurements, and to visualize the uptake and cell morphology after treatments, microscopic images of MonoMac-6 cells were captured. Washed and resuspended cells were plated on a 96-well flat-bottom tissue culture plate and images of the adherent cells were captured using an Olympus CKX41 microscope (Hamburg, Germany, equipped with Olympus U-RFLT50 mercury-vapor lamp, WideBlue DM500 BP460-490 BA520 IF filter, excitation wavelength range: 460–490 nm, objective: 20×).

### 4.6. In Vitro Cytotoxicity Determination by Colorimetric MTT-Assay

The in vitro cytotoxic effect of compounds was determined by MTT-assay (3-(4,5-dimethylthiazol-2-yl)-2,5-diphenyltetrazolium bromide assay). MonoMac-6 cells were plated into a 96-well plate with an initial cell number of 15 × 10^3^ per well. After 24 h of incubation at 37 °C, cells were treated for 16 h with the compounds, and the liposomal constructs dissolved in SFM RPMI-1640 medium (in the case of the compounds, SFM contained 2% DMSO). Control cells were treated for 16 h with SFM medium and SFM containing 2% DMSO. After incubation at 37 °C, cells were washed twice with SFM and the cell viability was determined by MTT. Then 45 μL MTT-solution (2 mg/mL) was added to each well. The respiratory chain and other electron transport systems reduce MTT and thereby form non-water-soluble violet formazan crystals within the cell. The amount of these crystals can be determined spectrophotometrically and serves as an estimate for the number of mitochondria and hence the number of living cells in the well [43,44,45,46,47].

After 4 h of incubation, cells were centrifuged for 5 min (1500 rpm, 863 g) and the supernatant was removed. The obtained formazan crystals were dissolved in 100 μL DMSO and the optical density (OD) of the samples was measured at 540 and 620 nm using ELISA Reader (iEMS Reader, Labsystems, Finland). OD620 values were subtracted from OD540 values. The percent of cytotoxic effect was calculated using the following equation: Cytotoxicity (%) = [1 − (OD_treated_/OD_control_)] × 100; where OD_treated_ and OD_control_ correspond to the optical densities of the treated and the control cells, respectively. In each case, two independent experiments were carried out with four parallel measurements. The 50% inhibitory concentration (IC_50_) values were determined from the dose-response curves. The curves were defined using OriginPro 2018 software.

### 4.7. Determination of In Vitro Antitubercular Efficacy of the Compounds on Mycobacterial Cultures and Infected Monocytes

In vitro antimycobacterial activity of the compounds on mycobacterial cultures was determined on slow-growing *Mtb* H_37_Rv (ATCC 27294) and *Mtb* A8 MDR strain (ATCC 35822, resistant to RIF and INH) [21] by serial dilution in Sula semisynthetic medium (prepared in-house, pH 6.5) [17,48]. Compounds were added to the medium as DMSO solutions at various doses (range of final concentration was between 0.05 and 100 μg/mL). MICs were determined after incubation at 37 °C for 28 days in the case of *Mtb* H_37_Rv, and for 28 days in the case of *Mtb* A8 MDR. MIC was the lowest concentration of a compound at which the visible inhibition of the growth of *Mtb* H_37_Rv and *Mtb* A8 MDR occurred. To confirm the growth inhibition, the colony forming unit (CFU) (the number of colonies that developed from the viable bacteria) was determined by sub-culturing onto drug-free Löwenstein-Jensen solid media [17,49]. Samples were incubated for a further 28 days in the case of *Mtb* H_37_Rv and *Mtb* A8 MDR. Experiments were repeated at least two times. 

To determine the activity on the intracellular bacteria-infected MonoMac-6 culture, a method based on our previous works [17] was applied. MonoMac-6 cell culturing before this experiment is the same as described in flow cytometry. Before infection, cells were seeded in a 24-well plate (2 × 10^5^ cells/1 mL medium/well) and incubated overnight. Adherent cells were infected with *Mtb* H_37_Rv culture at a multiplicity of infection (MOI) of ten for four hours. Non-phagocytosed extracellular bacteria were removed and the culture was washed three times with SFM RPMI. The infected monolayer was incubated for one day before antituberculous treatment. Infected cells were then treated with the two antitubercular agents in native (free compounds) and the two liposome-encapsulated environments (SUV_mixed_ and SUV_DPPC_) with serial dilutions. The highest concentration of the compounds on the cells was 100 μM. After three days, the treatment was repeated with a fresh solution of the compounds for an additional three days. Untreated cells were considered as a negative control. After the washing steps to remove the antitubercular agents, infected cells were lysed with 2.5% sodium dodecyl sulfate solution. The CFU of TB were enumerated on Löwenstein–Jensen solid media after six weeks of incubation.

## 5. Conclusions

Based on our measurements PEGylation and the encapsulation of TB501 and TB515 proved to enhance vesicular stability. TB501 and TB515 containing SUV_mixed_ liposomes presented improved homogeneity. The EE of TB515 showed superior results for both liposomal formulations, due to its chemical structure, which is more suitable for liposomal encapsulation compared to TB501. The cellular uptake of the pegylated pH-sensitive vesicles turned out to be just as efficient as the non-PEGylated SUV_DPPC_ liposomes paired with excellent nontoxicity and good intracellular inhibition in the case of the TB515 agent. Taking into consideration all circumstances in the case of intravenous administration the most promising drug formulation is the SUV_mixed_ encapsulated TB501.

## Figures and Tables

**Table 1:** The calculated EE of the two kinds of SUVs (SUV_mixed_ and SUV_DPPC_) for the two antitubercular agent candidates (TB501 and TB515) determined from Figure 3, (mean ± 3SE%).

Agent	SUV_mixed_ (%)	SUV_DPPC_ (%)
**TB501** (from data around 400 nm) (*n* = 15)	10.3 ± 0.5	13.6 ± 0.7
**TB515** (from data around 370 nm) *(n* = 12)	97.7 ± 1.7	98.2 ± 1.5

**Table 2:** Characterization of in vitro cytotoxicity for two antitubercular agent candidates (TB501 and TB515) on MonoMac-6 human monocytic cell line measured by flow cytometry (set on forward and side scattered (FSC, SSC); on two independent samples: duplicate); the ratio of living cells (%) with 160 μM agents for native agents and liposomal formulations (in SUV_mixed_ and in SUV_DPPC_). (Control means the untreated cells.)

Agent	Concentration (μM)	Native (%)	SUV_mixed_ (%)	SUV_DPPC_ (%)
**TB501**	0 (control)	90–92	89–92	91–93
	160	66–76	74–76	89–92
**TB515**	0 (control)	91–94	92–93	92–95
	160	77–82	82–90	85–90

**Table 3:** Characterization of the cellular uptake rate of two antitubercular candidates on MonoMac-6 human monocytic cells measured by flow cytometry (mean fluorescence intensity signal; FITC LP505; BP 530/30 in duplicates): uptake% at 160 μM agent concentration for TB501, and minimal agent concentrations (μM) of 100% uptake rate for TB515. For native agents and liposomal formulations (in SUV_mixed_ and SUV_DPPC_). (Concentration interval means the 100% uptake rate was reached between these two values in the serial dilutions.)

Agent	Native	SUV_mixed_	SUV_DPPC_
**TB501** (uptake% at 160 μM)	0.15–0.2	1.2–1.5	0.2–0.25
**TB515** (μM at 100% uptake)	20–40	80–160	80–160

**Table 4:** The number of colony forming units (CFUs) on *Mtb* H_37_Rv infected MonoMac-6 human monocytic cell cultures after treatments with different concentrations of the two antitubercular agent candidates (TB501 and TB515) in the native environment and in the two liposome-encapsulated (SUV_mixed_ and SUV_DPPC_) environments.

Agent	Concentration (μM)		Number of CFU	
	Untreated Control (0)	Confluent Colonies		
		Native	SUV_mixed_	SUV_DPPC_
**TB501**	12.5	>100	10–50	10–50
	25	50–100	0	1–10
	50	10–50	0	0
	100	1–10	0	0
**TB515**	12.5	>100	>100	>100
	25	>100	>100	>100
	50	>100	10–50	50–100
	100	>100	0	0

**Table 5:** The Minimum Inhibitory Concentration (MIC) of TB501 and TB515 agents on *Mtb* H_37_Rv and MDR A8 cultures. IC_50_ and SI values on MonoMac-6 cultures infected with *Mtb* H_37_Rv.

Agent	MIC * (µM)		MonoMac-6	
	*Mtb* H_37_Rv	MDR A8	IC_50_ (µM)	SI ** Mtb H_37_Rv
**TB501**	46	138	218	4.7
**TB515**	19	3.8	78	4.0

* MIC, γ of TB501 on *Mtb* H_37_Rv/MDR A8 = 20/60 µg/mL; of TB515 on *Mtb* H_37_Rv/MDR A8 = 5/1 µg/mL; ** selectivity index, SI = IC_50_ (µM)/MIC (µM).

**Table 6:** Summarized evaluation of antitubercular agent candidates (TB501 and TB515) from several aspects in native and in the two liposome-encapsulated (SUV_mixed_ and SUV_DPPC_) environment.

Agent	*Environment*	Long-Term Stability (SUV)	Encapsulation Efficiency	Cellular Uptake	Nontoxic	Intracellular Inhibition
**TB501**	***Native***	-	-	very low	moderate	moderate
	***SUV_mixed_***	good	low	low	moderate	excellent
	***SUV_DPPC_***	moderate	low	very low	excellent	excellent
**TB515**	***Native***	-	-	excellent	good	very low
	***SUV_mixed_***	excellent	excellent	good	excellent	good
	***SUV_DPPC_***	moderate	excellent	good	excellent	good

**Table 7:** Chemical features of the two in silico identified small molecular weight antitubercular agent candidates.

Agent	Hydrogen Bond Donor/Acceptor	Molecular Mass (g/mol)	log *P*
TB501	2/7	436.51	1.523
TB515	1/3	262.27	3.290
